# Global analysis of differential gene expression related to long-term sperm storage in oviduct of Chinese Soft-Shelled Turtle *Pelodiscus sinensis*

**DOI:** 10.1038/srep33296

**Published:** 2016-09-15

**Authors:** Tengfei Liu, Ping Yang, Hong Chen, Yufei Huang, Yi Liu, Yasir Waqas, Nisar Ahmed, Xiaoya Chu, Qiusheng Chen

**Affiliations:** 1 Laboratory of Animal Cell Biology and Embryology, College of Veterinary Medicine, Nanjing Agricultural University, Nanjing, Jiangsu Province, 210095, China

## Abstract

Important evolutionary and ecological consequences arise from the ability of female turtles to store viable spermatozoa for an extended period. Although previous morphological studies have observed the localization of spermatozoa in *Pelodiscus sinensis* oviduct, no systematic study on the identification of genes that are involved in long-term sperm storage has been performed. In this study, the oviduct of *P. sinensis* at different phases (reproductive and hibernation seasons) was prepared for RNA-Seq and gene expression profiling. In total, 2,662 differentially expressed genes (DEGs) including 1,224 up- and 1,438 down-regulated genes were identified from two cDNA libraries. Functional enrichment analysis indicated that many genes were predominantly involved in the immune response, apoptosis pathway and regulation of autophagy. RT-qPCR, ELISA, western blot and IHC analyses showed that the expression profiles of mRNA and protein in selected DEGs were in consistent with results from RNA-Seq analysis. Remarkably, TUNEL analysis revealed the reduced number of apoptotic cells during sperm storage. IHC and TEM analyses found that autophagy occurred in the oviduct epithelial cells, where the spermatozoa were closely attached. The outcomes of this study provide fundamental insights into the complex sperm storage regulatory process and facilitate elucidating the mechanism of sperm storage in *P. sinensis*.

Sperm storage in the female reproductive tract is defined as the retention of viable spermatozoa for an extended period of time^1^. It is typically used by a wide variety of animal species, including mammals, insects, fish, birds and reptiles, whose copulation is consistently asynchronous with ovulation^2^. Some mammalians and birds have evolved the capacity to store viable spermatozoa for only a few days or even hours, whereas some fish and reptiles can store spermatozoa for more than one year^3^,^4^. In reptiles, long-term sperm storage contributes to appropriately synchronize copulation, fertilization and nesting, which ensures the survival of these species^5^. Moreover, the storage of spermatozoa enables the females to control paternity through the selective use of spermatozoa^6^. Therefore, the elucidation of the molecular mechanism underlying sperm storage is beneficial for this species.

Considerable studies have suggested that the intimate contact between spermatozoa and the surface of oviduct epithelial cells is necessary to prolong spermatozoa survival, which is especially important for stimulating the synthesis of new proteins in the oviduct cells^7^,^8^. Furthermore, the oviduct cells have distinct secretory functions, providing nourishment and protection for the stored spermatozoa^9^. *In vitro*, binding to the oviduct epithelium prolongs spermatozoa survival and extends the motile life span in some species^10^. In reptiles, sperm-storage tubules (SSTs) in the oviduct can prevent sperm degeneration and maintain the potential fertilizing capacity of spermatozoa over long periods^11^. Recent studies have suggested that the microvillus blebs (MvBs) on the apical tips of SST epithelial cells contribute to the sustained sperm storage^12^. Understanding the relationship of spermatozoa and oviduct is a prerequisite to elucidate the mechanism of prolonged sperm storage.

Just as viviparity is regarded as having evolved in reptiles more than 100 times, the mechanisms of sperm storage have evolved multiple times^13^. Studies have shown the presence of anti-apoptotic proteins at the site of sperm storage in oviduct giving rise to speculation that this could be a functional component of sperm storage mechanisms^14^,^15^. Furthermore, other studies have suggested that immunity is an important mechanism underlying sperm storage. Low levels of cytokines during the period of sperm storage are thought to facilitate spermatozoa survival in hens^16^. More recently, some reports have shown that the long-term storage of spermatozoa is associated with autophagy, whose primary function is to degrade long-lived proteins and recycle cellular components^17^,^18^. Comprehensive dissection of the molecular regulatory mechanism of oviduct sperm storage *in vivo* will require further studies, which will contribute to further studies on spermatozoa conservation *in vitro*.

The Chinese soft-shelled turtle (*Pelodiscus sinensis*) belongs to a commercially important aquaculture reptile with high edible and pharmaceutical value in Asian countries such as China, Japan and Korea^19^. Spermatogenesis, copulation and ovulation are seasonal and segregational in turtles^5^. Our earlier studies clearly provided structural and ultrastructural evidence for sperm storage in the oviduct of female *P. sinensis*, and spermatozoa could survive in the oviduct during the hibernation season (from November to the following April)^9^,^20^. Recently, the draft genome sequences of *P. sinensis* have been published^21^, providing a useful database for genomic and functional investigations on some important biological traits in Chinese soft-shelled turtle. Next-generation sequencing (NGS) technologies have been proven to be an efficient and accurate choice for measuring gene expression under diverse biological conditions^22^. In particular, NGS-based RNA sequencing (RNA-Seq) has been widely employed for global gene expression profiling in some reptile species, including tuatara (*Sphenodon punctatus*), green anole lizard (*Anolis carolinensis*), garter snake (*Trimeresurus elegans*) and painted turtle (*Chrysemys picta*)^23^,^24^,^25^,^26^. Nevertheless, there is still limited knowledge concerning global gene expression and the mechanism underlying sperm storage in the *P. sinensis* oviduct.

In this study, RNA-Seq and digital gene expression (DGE) tag profiling from the *P. sinensis* oviduct during reproductive (July) and hibernation (January) seasons were performed using the Illumina HiSeq 2500 platform. The objectives of this study were to comprehensively identify the differentially expressed genes (DEGs) related to sperm storage and dissect the molecular mechanism underlying long-term sperm storage in *P. sinensis*. The critical genes involved in the immune response, apoptosis and autophagy regulation were isolated and characterized during sperm storage. The differential expressions of several DEGs related to sperm storage were validated by RT-qPCR, and their protein levels were detected by ELISA, western blot and IHC analysis. The number of apoptotic cells was detected by TUNEL analysis in July and January. Furthermore, morphological evidence further confirmed the storage of spermatozoa in the *P. sinensis* oviduct. These results will enhance our understanding of sperm storage in female *P. sinensis* and help elucidate the underlying mechanism in turtles.

## Results

### Sperm storage in the oviduct of *P. sinensis*

**Figure 1:**
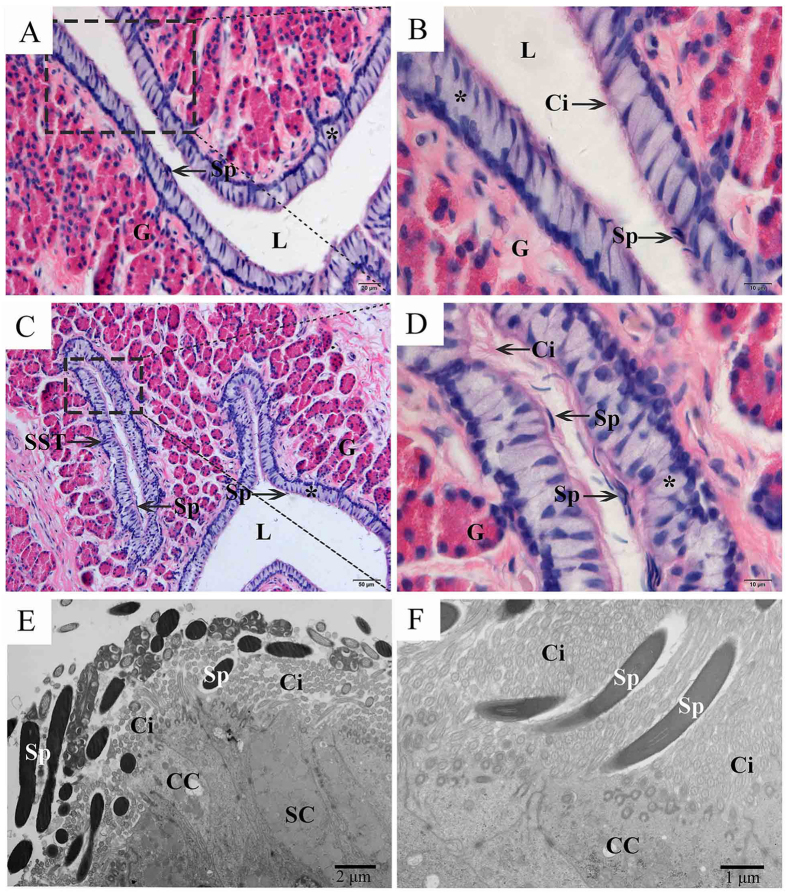
Distribution of spermatozoa in the oviduct of *P. sinensis* during the hibernation season (January), H&E staining and TEM. (**A**) Spermatozoa stored within the oviduct. (**B**) Spermatozoa were embedded among the cilia of the oviduct. (**C**) Spermatozoa stored in both the SST and lumen of the oviduct. (**D**) Spermatozoa were embedded among the cilia of SST. (**E**,**F**) Many spermatozoa were embedded among the cilia. Cilia (Ci), epithelium (*), gland cell (**G**), lumen (L), spermatozoa (Sp), secretory cell (SC), ciliated cell (CC). Scale bar = 50 μm (**C**), 20 μm (**A**), 10 μm (**B**,**D**), 2 μm (**E**) and 1 μm (**F**).

In *P. sinensis*, spermatogenesis and ovulation are out of phase with each other. After mating during the period from June to August, spermatozoa are stored in the oviduct from November to the following April (hibernation season)^27^. In this study, morphological analysis showed that spermatozoa were stored in the lumen (Fig. 1A,B) and SSTs (Fig. 1C,D) of the *P. sinensis* oviduct during the hibernation season (January). These spermatozoa were attached to the epithelial surface (Fig. 1B,D) and were primarily oriented with the heads toward epithelial cilia (Fig. 1E,F). Given our aims in global gene expression analysis during sperm storage, the oviduct of *P. sinensis* in July and January (of the following year) were chosen for RNA-Seq.

### RNA-Seq and identification of DEGs

**Figure 2:**
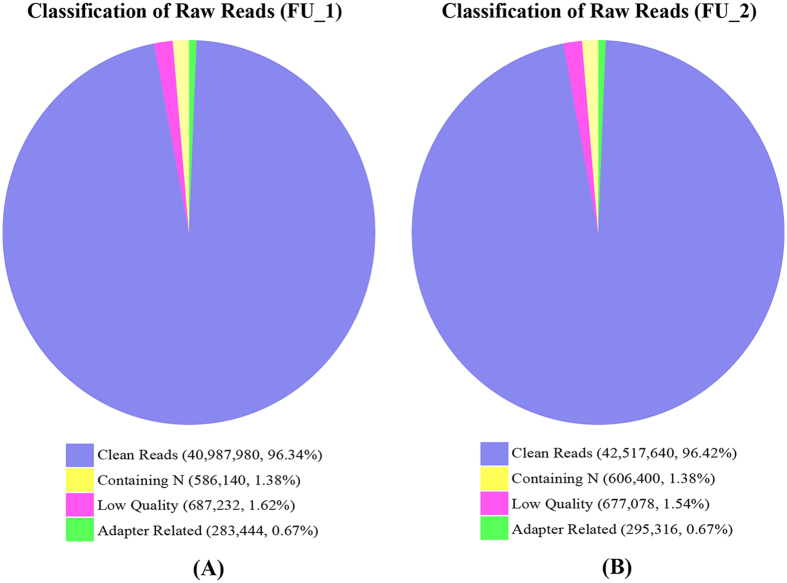
Classification of sequencing raw reads from the FU_1 (A) and FU_2 (B) libraries in *P. sinensis*.

**Figure 3:**
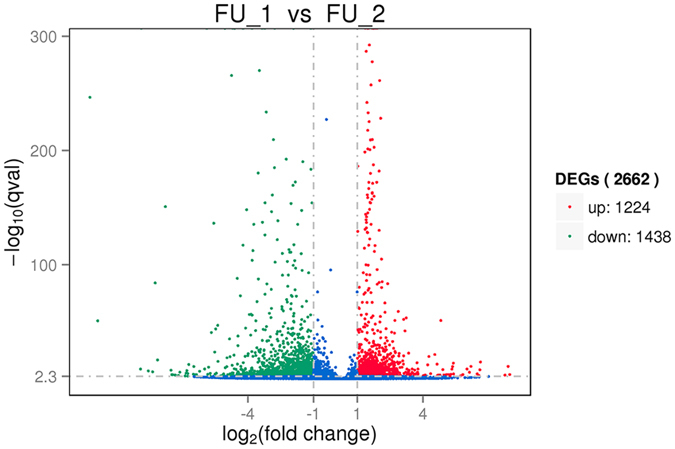
Scatter plot of differentially expressed genes (DEGs) between the FU_1 and FU_2 libraries in *P. sinensis*. The red and green dots represent up- and down-regulated genes in *P. sinensis*, respectively. The blue dots represent the genes without significant differential expression.

In this study, two cDNA libraries from the *P. sinensis* oviduct in July (FU_1) and January (FU_2) were constructed and sequenced using Illumina RNA-Seq technology. Approximately 42.5 and 44.1 million raw reads were obtained from the FU_1 and FU_2 libraries, respectively (Fig. 2). After removing the low-quality reads and adapter sequences, 40,987,980 and 42,517,640 clean reads were successfully obtained from the two libraries, among which 73.75% (30,229,347 in FU_1) and 69.85% (29,697,850 in FU_2) reads were mapped to the genome sequences of Chinese soft-shelled turtle (Table 1). To evaluate the gene expression levels, the FPKM method was used to calculate and normalize the read counts in the FU_1 and FU_2 libraries. The thresholds of |log_2_ (Fold change)| ≥ 1 and FDR ≤ 0.001 were used to determine DEGs. As a result, 2,662 DEGs were successfully identified, including 1,224 significantly up-regulated and 1,438 significantly down-regulated genes in the FU_2 library compared with the FU_1 library (Fig. 3).

### Functional annotation and enrichment analysis

**Figure 4:**
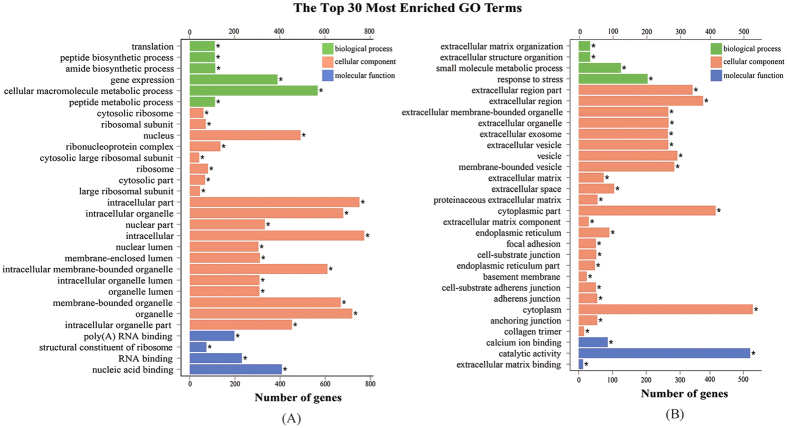
The top 30 most enriched Gene ontology (GO) terms (*P*-value ≤ 0.05) of up- (A) and down-regulated (B) DEGs in *P. sinensis.* Green, orange and blue represent the GO terms belonging to biological processes, cellular components and molecular functions, respectively.

GO classification and enrichment analyses with *P*-value ≤ 0.05 were performed according to three main ontologies: biological process, cellular component and molecular function. A total of 247 and 51 significantly enriched GO terms were obtained for the up- and down-regulated DEGs, respectively. The top 30 most enriched GO terms are shown in Fig. 4. Among these enriched terms, the most up-regulated genes were involved in some functional categories such as ‘binding’, ‘cellular component’, ‘cell part’, ‘cell’ and ‘cellular process’ (Supplementary Table S1), while the down-regulated genes were predominantly related to ‘single-organism process’, ‘organelle’, ‘membrane-bounded organelle’, ‘cytoplasm’ and ‘catalytic activity’ (Supplementary Table S2).

Pathway enrichment analysis with a *Q*-value ≤ 0.05 was implemented to assess the activated biological pathways during sperm storage. As a result, these DEGs were significantly enriched in seven and six KEGG pathways for up- and down-regulated genes (Supplementary Table S3), respectively. Among the up-regulated DEGs, the major enriched pathways were related to ‘Regulation of autophagy’ (pss04140), ‘Intestinal immune network for IgA production’ (pss04672), ‘Ribosome’ (pss03010) and ‘RNA transport’ (pss03013). The significantly enriched pathways for down-regulated DEGs were predominantly involved in ‘Apoptosis’ (pss04210), ‘p53 signaling pathway’ (pss04115), ‘MAPK signaling pathway’ (pss04010) and ‘mTOR signaling pathway’ (pss04150).

### Identification of critical DEGs involved in the sperm storage of *P. sinensis*

The RNA-Seq data and gene expression analysis showed several significantly DEGs involved in the immune response, apoptosis signalling pathway and autophagy regulation between reproductive and hibernation seasons (Supplementary Fig. S2). It is well known that the immunological response is a key component of the mechanism underlying sperm storage, and Toll-like receptors (TLRs) play important roles in innate immunity of the male reproductive tract^28^,^29^. In this study, DEGs were identified that were related to the Toll-like receptor (TLR) signalling pathway (pss04620) during sperm storage and included *TLR2*, *TLR4*, *CTSK*, *TRAF3*, *IRAK2*, *TOLLIP* and *MyD88* (Table 2; Supplementary Fig. S2A). Among them, *TRAF3*, *IRAK2* and *TOLLIP* were up-regulated, whereas *TLR2*, *TLR4*, *CTSK* and *MyD88* were down-regulated. Moreover, several proinflammatory cytokines such as *TNF*, *IL1B* (*IL1β*) and *IL18* were identified in the process of sperm storage and were significantly down-expressed during the hibernation season (Table 2; Supplementary Fig. S2A).

From the NGS-based RNA-Seq analysis, the apoptosis signalling pathway (pss04210) was identified as the most frequently used pathway related to sperm storage. As a result, several genes were found to be related to the pathway of apoptosis, such as *MCL1* and *BCL2L11* belonging to the Bcl-2 family, *CASP6* and *CASP9* from the Caspase family, and *BAG4* and *BAG5* encoding BAG1-related protein (Table 3; Supplementary Fig. S2B). Moreover, the p53 signalling pathway (pss04115) plays an important role in the induction of apoptosis^30^, and some differentially regulated genes were identified to be involved in this pathway (Supplementary Fig. S2C). Importantly, most of the p53 signalling-related genes were down-regulated, such as *PERP*, *CytC*, *PIDD* and *SCOTIN* (Table 3), indicating that the regulation of the expression of these genes may be crucial for modulating the network of sperm storage.

Furthermore, our results showed that many pathways were involved in the regulation of sperm storage, and the regulation of autophagy (pss04140) appears to be most significant in the pathway enrichment analysis. In this study, some autophagy-related genes such as *ATG16L1*, *ATG14*, *ATG5* and *ATG12* were found to be differentially expressed (Table 4; Supplementary Fig. S2D). In addition, *MAP1LC3B* (*LC3B*) encoding microtubule-associated protein 1 light chain 3 beta, and *BNIP3*, belonging to the pro-cell death member of the Bcl-2 family, were significantly up-regulated during the hibernation season.

### Expression profile analysis by RT-qPCR

**Figure 5:**
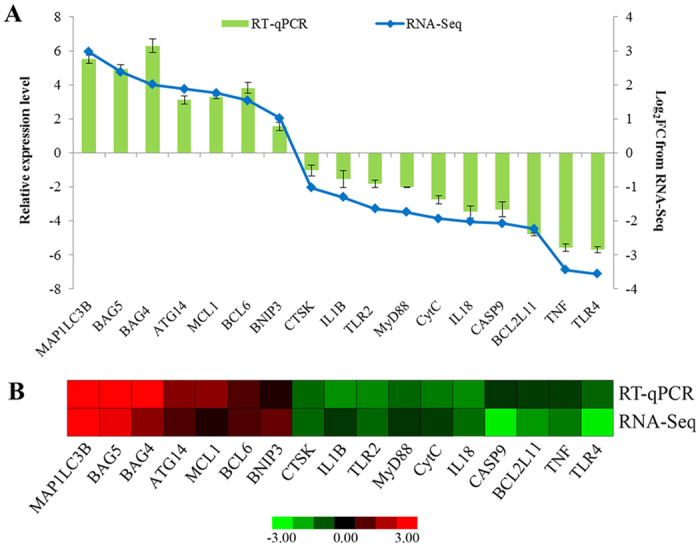
Expression validation of selected DEGs related to sperm storage in *P. sinensis*. (**A**) The relative expression levels of DEGs by RT-qPCR (green bar) were compared with the transcript abundances from RNA-Seq (blue bar). (**B**) Heat map diagram of the expression patterns of DEGs in *P. sinensis*. The red and green colours indicate up- and down-regulated genes in the FU_2 library compared with those in the FU_1 library, respectively.

To validate the differential expression patterns of DEGs during sperm storage in *P. sinensis*, 17 genes were randomly selected and subjected to RT-qPCR analysis. These selected genes included 7 immune-response genes (including *CTSK*, *IL1B*, *TLR2*, *MyD88*, *IL18*, *TNF* and *TLR4*), 7 apoptosis-related genes (including *BAG5*, *BAG4*, *BCL6*, *CytC*, *CASP9*, *BCL2L11* and *MCL1*) and 3 genes (including *MAP1LC3B*, *ATG14* and *BNIP3*) related to autophagy regulation. Moreover, the relative expression levels of these genes determined by RT-qPCR analysis were compared with the read counts calculated by FPKM method. The results revealed that these gene expression patterns were in agreement with the transcript abundance changes from RNA-Seq (Fig. 5), indicating the highly accuracy and quality of DGE sequencing and expression analysis.

### Circulating TNFα, IL1β and IL18 concentrations in oviduct tissue

**Figure 6:**
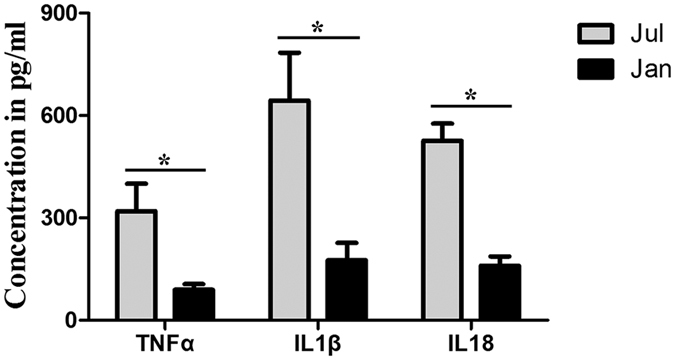
Concentration of circulating TNFα, IL1β and IL18 in oviduct tissue of *P. sinensis*. The values (*) obtained in July were significantly higher (*P* < 0.05) than those obtained in January.

To further detect the protein levels of three immune response-related genes such as TNFα, IL1β and IL18, the concentrations of circulating TNFα, IL1β and IL18 in the oviduct of *P. sinensis* were detected during the reproductive and hibernation seasons using ELISA in this study. The results showed that the circulating TNFα, IL1β and IL18 concentrations were significantly lower (*P* < 0.05) in January than in July (Fig. 6), which is consistent with their down-expression patterns by DGE analysis. These results implied that the decreased immune reaction may be in favor of the long-term sperm storage in female *P. sinensis*.

### Evaluation of cell apoptosis by TUNEL

**Figure 7:**
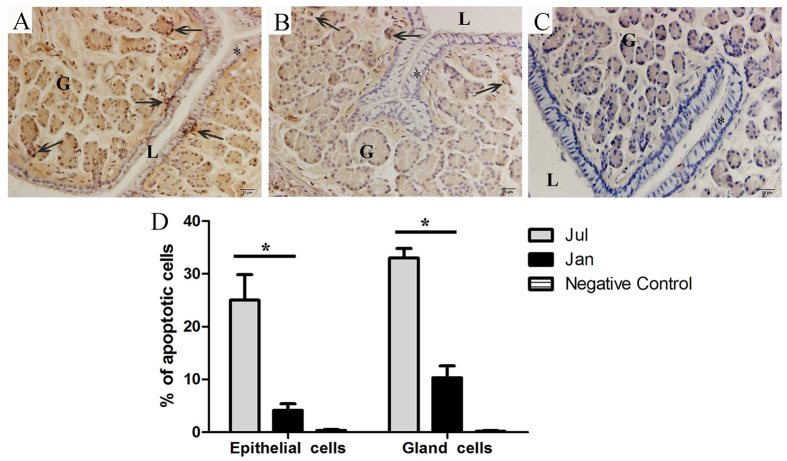
Representative photographs of TUNEL staining in July (A), January (B), negative control (C) of the oviduct, and the counting of the TUNEL positive cells (D). Epithelium (*), gland cell (G), lumen (L), TUNEL positive cells (↑). Scale bar = 20 μm. The values (*) obtained in July were significantly higher (*P* < 0.05) than those obtained in January.

The effect of stresses imposed by seasonal change on *P. sinensis* oviduct apoptosis was revealed by *in situ* 3’-OH end tailing of fragmented DNA. In July, many TUNEL-positive cells were observed (Fig. 7A). Most of the TUNEL-positive cells were oviduct epithelial cells and gland cells (Fig. 7A). In January, the number of apoptotic cells was substantial decreased (Fig. 7B), which is consistent with the down-regulated enriched pathways of apoptosis and p53 signaling. No labelling was seen in negative control sections (Fig. 7C). These results suggested that the number of apoptotic cells were decreased during the sperm storage in female *P. sinensis*.

### Expression and localization of autophagy protein LC3B in the *P. sinensis* oviduct

**Figure 8:**
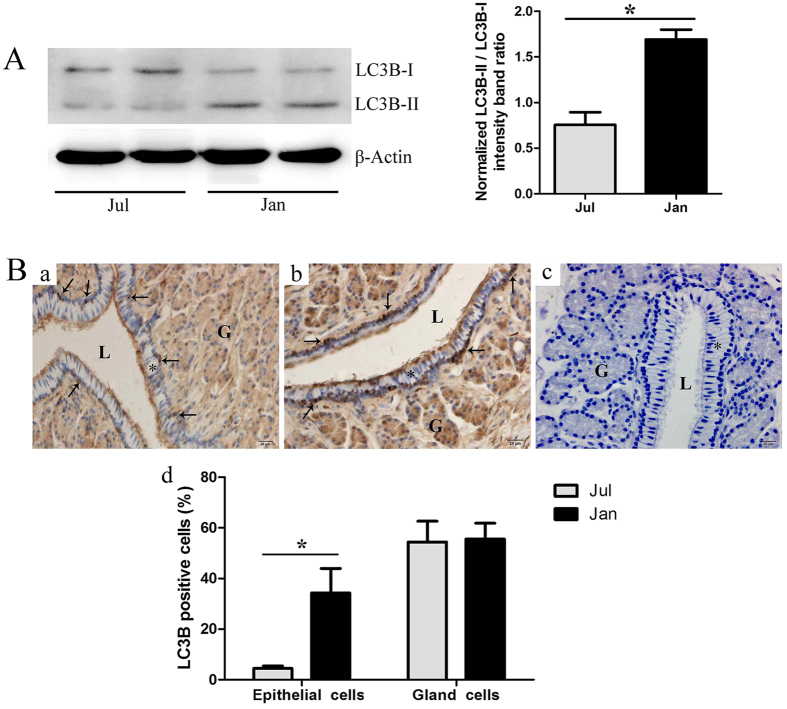
LC3B protein expression in the *P. sinensis* oviduct during the reproductive (July) and hibernation (January) seasons. (**A**) Western blot analysis of LC3B protein expression. The histogram represents densitometric analysis of the immunoblots. (**B**) Immunohistochemical localization of LC3B. (a) July, (b) January, (c) negative control and (d) the quantification of LC3B positive cells. Epithelium (*), gland (G), lumen (L), LC3B positive cells (↑). Scale bar = 20 μm. The values (*) obtained in January were significantly higher (*P* < 0.05) than those obtained in July.

Western blot was performed to validate the ratio of LC3B-II/LC3B-I in the oviduct of *P. sinensis*, with *β-actin* as an internal control. As shown in Fig. 8A, protein bands immunopositive for the LCB3-I and LCB3-II forms were clearly evident in each sample. Compared with July, the ratio of LC3B-II/LC3B-I was significantly increased (*P* < 0.05) in January, indicating that the autophagy level was much higher in the period of sperm storage.

Immunohistochemistry (IHC) also showed an LC3B-positive reaction in the oviduct (Fig. 8B). Immunostaining was primarily observed in the cytoplasm of oviduct epithelial cells (including ciliated cells and secretory cells), whereas no staining was detected in the negative control sections (Fig. 8Bc). The results showed that autophagy was found in the oviduct epithelial cells where the spermatozoa intimately contact.

### Numerous autophagosomes were observed by TEM within oviduct epithelial cells during the hibernation season

**Figure 9:**
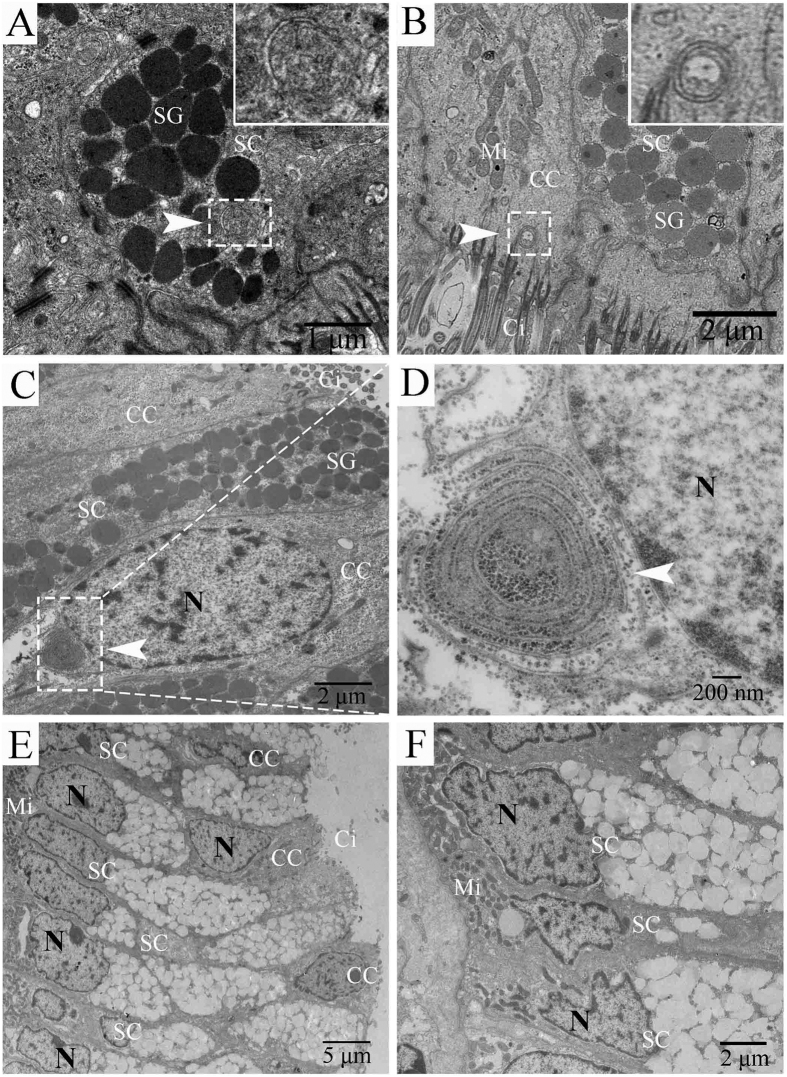
TEM photograph of oviduct epithelial cells in January (A–D) and July (E,F). (**A**,**F**) secretory cells. (**B**–**D**) ciliated cells. (**E**) secretory and ciliated cells. Autophagosome (white arrow), secretory cell (SC), ciliated cell (CC), cilia (Ci), secretory granules (SG), mitochondria (Mi), nucleus (N). Scale bar = 5 μm (**E**), 2 μm (**B**,**C**,**F**), 1 μm (**A**) and 200 nm (**D**).

To determine whether autophagy occurs in the oviduct of *P. sinensis*, we observed the ultrastructural morphology of the oviduct by TEM in January and July. In January, double- and multi-membrane-bound autophagic vesicles containing undigested cytoplasmic material were observed within the oviduct epithelial cells (Fig. 9), typical features of autophagosomes immediately prior to lysosomal fusion. In addition, these cells included ciliated cells (Fig. 9B,C) and secretory cells (Fig. 9A). Moreover, in July, there are no typical autophagosomes were observed in the oviduct epithelial cells (Fig. 9E,F). These results are consistent with those of LC3B immune staining (Fig. 8B).

## Discussion

The female reproductive tract plays a major role in the stringent selection of spermatozoa, regulation of spermatozoa motility and survival of spermatozoa^31^. Recent studies have elucidated that the interaction between spermatozoa and oviduct epithelial cells could prolong the life span of stored spermatozoa in *P. sinensis*^20^,^27^. The evolved specialized SSTs can maintain spermatozoa viability for a long duration in some female reptiles, including turtles^9^,^11^. In the current study, the stored spermatozoa were found in both the lumen and SSTs of the *P. sinensis* oviduct during the hibernation season (January), suggesting that the oviduct might provide a suitable microenvironment to maintain spermatozoa for long periods in *P. sinensis*. Although morphological evidence has clearly confirmed the localization of spermatozoa in the female reproductive tract, the molecular mechanisms underlying long-term sperm storage in the oviduct of turtles remain obscure. In this study, two *P. sinensis* oviduct cDNA libraries during the reproductive and hibernation seasons were constructed and sequenced using RNA-Seq technology, respectively. A list of DEGs related to the immune response, apoptosis and autophagy signalling pathways were identified and comprehensively profiled. To our knowledge, this is the first research on the large-scale identification and profiling of sperm storage-related genes in *P. sinensis*.

### Immune-response genes related to prolonged sperm storage

Spermatozoa and seminal proteins are antigenic to the female immune system^32^. However, the stored spermatozoa in the oviduct for long-time survival seem to be tolerated by the female. Increasing studies across diverse taxa have discussed the relationship between the immunological response in the reproductive tract and sperm storage^33^,^34^. Previous studies in insects have shown that a reduction in immune function was necessary for the prolonged sperm storage in females^28^, whereas the quality of stored spermatozoa decreased following an immune insult^35^. Mcnamara *et al*.^36^ suggested that a significant immune challenge resulted in a reduction in the viability of stored spermatozoa in female *Teleogryllus oceanicus*. Atikuzzaman *et al*.^37^ reported that the spermatozoa surviving in the SSTs of hens require the differential expressions of specific genes, and most immune-reactive genes were down-regulated. In the present study, many genes implicated in the TLR signalling pathway were significantly differentially expressed during the hibernation season compared with the reproductive season. The TLR signalling pathway is responsible for modulating the innate immune responses in vertebrates against parasites, bacteria and viruses^38^. The TLR signal pathway cascade could be activated as soon as specific ligand bind to *TLRs*, inducing the release of inflammatory cytokines and chemokines^39^. The overexpression of the TLR2, 4 genes in human spermatozoa reduced the motility of spermatozoa and impaired the potential for fertilization significantly^40^. Our previous study demonstrated the distribution of TLR2, 4 in the oviduct of *P. sinensis* and inferred the possible role of TLR2, 4 in sperm storage^41^. In this study, the expressions of several transcripts encoding *TLR2*, *TLR4*, *CTSK* and *MyD88* were significantly decreased during the hibernation season, suggesting that TLR cascades contributed to the tolerance of the stored spermatozoa in response to immune reaction in female *P. sinensis*.

Moreover, the other immune response, including *TNF*, *IL1β* and *IL18*, were found to be down-expressed during the hibernation season. *TNF*, as an inflammatory cytokine, is mainly produced by macrophages and monocytes during acute inflammation^42^. A previous study revealed that *TNF* has cytotoxic activity to cause germ cell apoptosis in mammalian cells^43^, inferring that the down-expression of *TNF* may be responsible for the stored spermatozoa in the oviduct of *P. sinensis*. Another inflammatory mediator, *IL1β*, could enhance the immune response induced by various stimuli, including mitogens, cytokines and microbial products^44^. Das *et al*.^45^ reported that the changes in mRNA expression of *IL1β* and *TNF*-related molecules were significant for spermatozoa survivability in hens, and the decrease in *IL1β* may permit spermatozoa to survive in SSTs. *IL18*, also known as IFN-inducing factor, is involved in inflammation, ischemic tissue injury and T-cell-mediated immunity^46^,^47^. The increased levels of *IL1β* and *IL18* were correlated with the rate of spermatozoa with abnormal morphology in humans^48^. In this study, the down-expression of *TNF*, *IL1β* and *IL18* during the hibernation season was validated by RT-qPCR and ELISA analyses. These results indicated that these immune-related genes may modulate the immune response in *P. sinensis* to tolerate the presence of allogeneic spermatozoa in the oviduct for lengthy periods.

### Down-regulation of apoptosis genes may be in favour of long-term sperm storage

Apoptosis is a physiological programmed cell death process that plays an essential role in the process of gamete maturation and embryogenesis^49^. Studies have indicated that apoptosis, as the regulatory mechanism of germ cell death, is implicated in long-term sperm storage and plays vital roles in the removal of spermatozoa without viability from female reproductive tracts^50^,^51^. Previous study showed that the oviduct cells can secrete several apoptosis-related proteins to lumen, and these proteins could interact with the stored spermatozoa during the period of sperm storage^52^. Urhausen *et al*.^15^ reported that the expression of apoptosis-related proteins was investigated in the dog oviduct, suggesting that the control of apoptosis may be a major functional component of the mechanism underlying sperm storage prior to fertilization. Furthermore, in female *Scotophilus heathi*, the interplay between pro- and anti-apoptotic factors was shown to hold the key to prolonged sperm storage^53^. Recently, our direct evidence revealed that the anti-apoptosis might enable the oviduct to maintain the storage of spermatozoa in *P. sinensis*^14^. These findings suggest that the apoptosis of oviduct cells is closely related to the long-term spermatozoa survival in females.

In the present study, using TUNEL, which is a routine and normal method to detect apoptosis^54^, more apoptotic cells were detected in July than in January (Fig. 7). Moreover, some up-regulated anti-apoptosis genes and down-regulated pro-apoptosis genes were identified during sperm storage by functional annotation and expression analysis, indicating that these gene expression alterations might contribute to long-term sperm storage in *P. sinensis*. Among these up-regulated anti-apoptosis genes, *BCL6* encodes a Kruppel-type zinc finger transcriptional repressor, and its overexpression was found to obviously inhibit spermatozoa apoptosis in response to various stressors^55^. *MCL1*, another member of the anti-apoptotic Bcl-2 family, is an early-response gene in the apoptotic signalling cascade and exerts its function in delaying apoptosis under apoptosis-inducing conditions^56^. The functions in anti-apoptotic activities were also shared by *BAG4* and *BAG5*, which belong to the Bcl-2-associated athanogene (BAG)-family. Thus, the up-expression of these anti-apoptosis genes, including *BCL6*, *MCL1*, *BAG4* and *BAG5*, may repress the apoptotic effect in the oviduct and thereby protect the resident spermatozoa in female *P. sinensis*. Moreover, in this study, the participation of down-regulated pro-apoptosis genes such as *BCL2L11*, was also found during sperm storage. *BCL2L11* (also known as BIM) could promote the release of apoptogenic proteins and inactivate the anti-apoptosis BCL-2 proteins to trigger apoptosis^57^. These findings indicated that the down-regulated pro-apoptosis genes negatively control the initiation of apoptosis in *P. sinensis*.

It has been extensively shown that the p53 signalling pathway could induce DNA damage-triggered apoptosis in various cell types^30^. The effects of oxidative stress are particularly important during sperm storage either by cooling or *in vivo*, and the reactive oxygen species (ROS) was suggested as a main factor in the inhibition of spermatozoa longevity^58^,^59^. Previous studies have demonstrated that the decreased levels of ROS could suppress the release of CytC from mitochondria to the cytosol, promoting the increase in spermatozoa longevity in the female reproductive system for diverse organisms^58^,^59^. The CytC protein could bind to Apaf-1 and, in turn, activate CASP9, triggering apoptosis^60^. Moreover, the ROS-CytC-Caspase axis in the p53 pathway was suggested to play key roles in apoptosis^61^. Here we found that the two components of the p53-dependent apoptosis pathway, *CytC* and *CASP9*, were down-regulated during the hibernation season. Taken together, these findings implied that the oviduct contributing to prolong sperm storage in *P. sinensis* might suppress oxidative stress-induced apoptosis through the ROS-CytC-Caspase model in the p53 pathway.

### Regulation of autophagy involved in sperm storage

Autophagy is a highly conserved catabolic process that is primarily responsible for the nonspecific degradation of redundant and recyclable cellular components^62^. In the process, portions of the cytoplasm, damaged proteins and organelles are sequestered in double- or multi-membrane structures called autophagosomes, which deliver material to lysosomes for digestion^63^. Recently, increasing reports have focused on the involvement of autophagy in spermatozoa. In *Caenorhabditis elegans*, fertilizing spermatozoa trigger the recruitment of autophagosomes and subsequent paternal mitochondria degradation to prevent paternal mitochondrial DNA transmission^64^. The expression of autophagy-related proteins in stallion spermatozoa suggested that autophagy is the main strategy for the survival of cooled stored spermatozoa^17^. Moreover, a series of autophagy-related genes (ATGs) was implicated in the regulation of autophagy. For instance, *ATG14* is a critical element of autophagic initiation and can interact with phosphatidylinositol 3-phosphate in the bilayer membrane during autophagosome formation^65^. The overexpression of *ATG14* has been demonstrated to enhance autophagic activity in yeast and mammals^63^,^66^. In addition, *BNIP3*, as a pro-cell death member of the Bcl-2 family, was found to inhibit the mTOR (mechanistic target of rapamycin) pathway and promote autophagy^67^. The knockdown of *BNIP3* was shown to inhibit autophagy and promote the necrotic cell death of tumour cells^68^. In this study, the up-expression of *ATG14* and *BNIP3* was detected in the process of sperm storage, implying that these genes might promote autophagy during the hibernation season and thereby contribute to prolong the life span of stored spermatozoa in *P. sinensis*.

Furthermore, NGS-based expression analysis and RT-qPCR detected the increased transcript abundance of the *LC3B* gene, and western blot analysis further revealed the high ratio of LC3B-II/LC3B-I in the *P. sinensis* oviduct during the hibernation season. The microtubule-associated protein 1 light chain-3β (LC3B) exists in two forms: LC3B-I and LC3B-II. The unprocessed form of LC3B is cleaved by ATG4 into a cytosolic form, known as LC3B-I^63^. Upon induction of autophagy, LC3B-I is processed to LC3B-II, which is inserted into both the inner and outer membranes of the growing autophagic vesicle^69^. The conversion from LC3B-I to LC3B-II is a cellular readout of autophagy levels^70^. Therefore, our observations indicated that autophagy was increased during the sperm storage period in *P. sinensis*. Moreover, IHC showed that LC3B-positive cells were mainly located along the oviduct epithelium, in which ciliated cells and secretory cells are distributed. Furthermore, under TEM, autophagosomes containing undigested cytoplasmic material were observed and distributed within the ciliated cells and secretory cells, indicating that autophagy can occur in the oviduct epithelium when spermatozoa are stored during the hibernation season. It is well known that autophagy serves as an alternative energy source to sustain cellular function under stress^71^. A sufficient energy source at the site of sperm storage is essential for the prolonged survival of spermatozoa^72^. Furthermore, H&E staining and TEM analysis demonstrated that the spermatozoa were closely attached to the oviduct epithelium during sperm storage (Fig. 1). Hence, it is reasonably inferred that autophagy could provide available energy for the oviduct epithelial cells to support the stored spermatozoa in the female *P. sinensis*.

## Methods

### Animals

In this study, any work involving experimental animals was conducted according to the guidelines of the Animal Research Institute Committee of Nanjing Agriculture University, China. The experimental protocol and study were approved by the Science and Technology Agency of Jiangsu Province. The approval ID is SYXK (SU) 2010–0005. All efforts were made to minimize the animal’s suffering. Ten adult female *P. sinensis* in sex mature stage, from Yangcheng Lake in Suzhou (31°N, 120°E), southeastern China, were used in this study. The animals were anesthetized by sodium pentobarbital (20 mg/kg) administered intraperitoneally and were subsequently sacrificed through cervical dislocation. They were slaughtered in July (reproductive season) and January (hibernation season) with five turtles at each time. The oviduct of *P. sinensis* can be divided into five segments: infundibulum, magnum, isthmus, uterus, and vagina. The spermatozoa were predominantly stored in the anterior parts of the oviduct (including the vagina, uterus and isthmus)^27^. Therefore, the anterior parts of the oviduct were used for analysis in this study. One portion of the oviduct samples of each turtle was collected and immediately fixed for light and transmission electron microscopy (TEM), respectively. The other portion of the oviduct samples was ground in liquid nitrogen immediately and stored at −80 °C for gene expression analysis.

### Haematoxylin-eosin (H&E) staining

The samples were embedded in paraffin after fixation in neutral-buffered formalin for 48 h, and serially sectioned (6 μm). The sections were stained with haematoxylin and counter-stained with eosin (haematoxylin for 1 min and 1% eosin for 10 sec) for observation under a light microscope (BX53; Olympus; Tokyo, Japan).

### Transmission electron microscopy (TEM)

The tissues was cut into small sections (1 mm^3^) and immersed in a mixture of 2.5% glutaraldehyde fixative in phosphate-buffered saline (PBS) (4 °C, pH 7.4, 0.1 M) for 24 h, followed by post-fixation in similarly buffered 1% osmium tetroxide for 1 h at room temperature. Subsequently, the samples were dehydrated in ascending concentrations of ethyl alcohol, infiltrated with a propylene oxide-Araldite mixture and then embedded in Araldite. Ultrathin sections (50 nm thickness) were stained with uranyl acetate and lead citrate. The sections were examined and photographed using JEM-1200EX TEM.

### RNA isolation, library construction and sequencing

Equal amounts of oviduct samples from five turtles in July and January were pooled for cDNA library construction. Total RNA was extracted using TRIzol reagent (Invitrogen, Carlsbad, CA, USA) according to the manufacturer’s protocol and was incubated with 20 U/ml DNaseI (Sigma-Aldrich, St. Louis, MO, USA) for 15 min at 37 °C to remove genomic DNA contamination. Next, the RNA quality and quantity were examined using a NanoPhotometer spectrophotometer (IMPLEN GmbH, Munich, Germany) and an Agilent 2100 Bioanalyzer (Agilent Technologies, CA, USA). The RNA integrity number (RIN) of all samples was greater than 8.0. Two cDNA libraries were prepared using the NEBNext Ultra RNA Library Prep Kit for Illumina (NEB, USA) following the manufacturer’s recommendations. Briefly, mRNAs were enriched using magnetic beads with Oligo (dT) (Life Technologies, CA, USA) and then were fragmented into small pieces with NEBNext first-strand synthesis reaction buffer under an elevated temperature. First-strand cDNA was synthesized with random hexamer primers using M-MuLV Reverse Transcriptase Buffer (New England BioLabs, Whitby, ON). Second-strand cDNA synthesis was subsequently performed using DNA Polymerase I and RNase H. Thereafter, the cDNA was purified using the Qiaquick PCR extraction kit (Qiagen, Valencia, CA, USA) and subjected to end reparation and poly (A) tail addition. Suitable fragments were enriched by PCR amplification. The two libraries were sequenced using an Illumina HiSeq 2500 platform. The sequence data were deposited in NCBI Sequence Read Archive (SRA, http://www.ncbi.nlm.nih.gov/Traces/sra/) with accession numbers of SRX1675543 (FU_1) and SRX1672963 (FU_2).

### Analysis of sequencing data

Raw data were filtered to remove both the reads containing adapter and ploy-N and the low-quality reads (Q < 20) generating the clean data. For sequence mapping, clean reads were aligned to the Chinese soft-shelled turtle genome sequence^21^ using TopHat v2.0.9, allowing no more than two base mismatches. The read counts of each gene were summarized by HTSeq v0.6.1 and adjusted using the edgeR program package through one scaling normalized factor. The DEGSeq R package (1.12.0) was used to detect DEGs. The expression of each gene was normalized by the reads per kilobase per million mapped reads (RPKM) among different samples. We judged the significant differences in gene expression using the threshold value of |log_2_ (Fold change)| ≥ 1 with a False Discovery Rate (FDR) ≤ 0.001 and *P*-value ≤ 0.005. The fold change was calculated as follows: Fold change = RPKM in FU_2/RPKM in FU_1.

### Enrichment analysis of differentially expressed genes

Gene ontology (GO) enrichment analysis of DEGs was performed using they GOseq R package, in which gene length bias was corrected. GO terms with a corrected *P*-value ≤ 0.05 were considered significantly enriched by DEGs. Pathway analysis was mainly based on the Kyoto Encyclopaedia of Genes and Genomes (KEGG, http://www.kegg.jp/kegg/pathway.html) database^73^. The KOBAS software was applied to test the statistical enrichment of differential expression genes in KEGG pathways.

### RT-qPCR validation

The RNA extractions from *P. sinensis* oviduct in July and January were used for RT-qPCR analysis. Primers for RT-qPCR were designed using Beacon Designer 7.0 software (Premier Biosoft International, USA) and are listed in Supplementary Table S5. Total RNA samples were reverse transcribed into cDNAs using the SuperScript First-Strand Synthesis System (Invitrogen, Carlsbad, CA, USA). RT-qPCR was performed according to previous reports^20^. The relative gene expression levels were calculated using the 2^−ΔΔ*C*T^ method with *β-actin* gene as the internal control. Three biological replicates were performed for each gene.

### Enzyme-linked immunosorbent assay (ELISA)

Oviduct tissues were collected, and total proteins were extracted using a protein extraction kit (Thermo Fisher Scientific, Rockford, USA). Protein concentrations were measured using the Bradford method (BioRad Laboratories, CA, USA). TNFα, IL1β and IL18 in oviduct tissue homogenates were detected by ELISA using commercially available kits (KA0191, KA0356, KA0561; Abnova, Walnut, CA, USA). The pooled oviduct tissue homogenates were serially diluted and tested against a standard curve. Diluted samples running parallel to the standard curve indicated the validity of this assay in *P. sinensis*. The ELISA was performed according to the manufacturer’s instructions. All of the samples and standards were measured in duplicate.

### TdT-mediated dUTP-biotin nick end labelling assay (TUNEL)

Oviduct sections were treated with 20 mg/mL proteinase K (Sigma-Aldrich, St. Louis, MO, USA) for 20 min at room temperature. Next, they were heated (60 °C) for 30 min in 0.1 M citrate buffer (pH 6.0), allowed to cool for 15 min, exposed to 3% H_2_O_2_ in water (to quench endogenous peroxidase activity) for 15 min, and rinsed thrice in distilled water (5 min/rinse). Staining was performed using a commercially available apoptosis detection kit (ApopTag Peroxidase *In Situ* Apoptosis Detection Kit, Millipore, Billerica, MA) following the manufacturer’s instructions. The TUNEL reaction mixture was incubated on the slides for 60 min at 37 °C. A positive signal was detected using diaminobenzidine (Vectorlabs, CA, USA). The slides were counterstained with haematoxylin before being mounted in neutral balsam (Sigma-Aldrich, St. Louis, MO, USA). Negative controls were processed in an identical manner, except that the TdT enzyme was replaced with the same volume of distilled water. From each specimen, three sections were initially examined under light microscopy at low magnification (×100). Five fields per section were randomly examined at a higher magnification (×400). Two investigators examined the samples microscopically in a blinded fashion. The percentage of the TUNEL positive cells was used to determine the apoptosis rate^74^.

### Western blot

Western blot was performed as described previously^20^ using equal amounts of protein (40 μg/lane) from the oviduct. Anti-LC3B antibody (ab48394, Abcam Inc., Cambridge, MA, USA) was used at a concentration of 2 μg/ml, and protein band detection was performed using an ECL detection system (Vazyme Biotech, China). The validity of the anti-LC3B antibody in *P. sinensis* was determined by negative and positive control analysis (Supplementary Fig. S1).

### Immunohistochemistry (IHC)

Immunohistochemistry staining was performed according to a previously described protocol^14^. Briefly, the sections were deparaffinized in xylene, cleared in an ethanol series, blocked with 3% H_2_O_2_ in methanol, and heated for 30 min in sodium citrate buffer (pH 6.0). The primary anti-LC3B antibody (ab48394, Abcam Inc., Cambridge, MA, USA) at a 1:200 dilution was incubated with the sections overnight at 4 °C. Negative controls were incubated with a non-specific anti-rabbit IgG. Biotinylated secondary antibody and Vector ABC reagent (Vector Laboratories, Burlingame, CA) were subsequently added according to the manufacturer’s instructions. After washing, the sections were incubated with FAST DAB Peroxidase Substrate (Sigma-Aldrich, St. Louis, MO, USA) and counterstained with haematoxylin for 30 sec. The slides were analysed under a light microscope and photographed. The LC3B positive cells were automatically counted at ten randomly selected fields per sample by using the Image-Pro Plus (IPP) software, version 6.0 (Media Cybernetics, Bethesda, MD, USA)^75^.

### Statistical analysis

The data were expressed as the means ± SEM and were analyzed using SPSS software version 14.0 using an independent t-test. *P*-values less than 0.05 were considered to be statistically significant.

## Additional Information

**How to cite this article**: Liu, T. *et al*. Global analysis of differential gene expression related to long-term sperm storage in oviduct of Chinese Soft-Shelled Turtle *Pelodiscus sinensis*. *Sci. Rep.* **6**, 33296; doi: 10.1038/srep33296 (2016).

## Supplementary Material

Supplementary Information

Supplementary Dataset 1

Supplementary Dataset 2

Supplementary Dataset 3

Supplementary Dataset 4

Supplementary Dataset 5

## Figures and Tables

**Table 1:** Overview of DGE sequencing and mapping.

Sample name	FU_1	FU_2
Clean reads	40,987,980	42,517,640
Total mapped	30,229,347 (73.75%)	29,697,850 (69.85%)
Multiple mapped	673,618 (1.64%)	745,832 (1.75%)
Uniquely mapped	29,555,729 (72.11%)	28,952,018 (68.09%)
Read-1	15,333,275 (37.41%)	14,993,354 (35.26%)
Read-2	14,222,454 (34.7%)	13,958,664 (32.83%)
Reads map to ‘+’	14,764,284 (36.02%)	14,527,366 (34.17%)
Reads map to ‘−’	14,791,445 (36.09%)	14,424,652 (33.93%)
Non-splice reads	16,943,967 (41.34%)	18,532,194 (43.59%)
Splice reads	12,611,762 (30.77%)	10,419,824 (24.51%)

**Table 2:** DEGs involved in the immune response during sperm storage.

Gene ID	Log_2_ Fold change	Associated Gene Name	Interpro Description
up-regulated genes			
ENSPSIG00000003573	3.1836	PIGR	Immunoglobulin subtype
ENSPSIG00000015342	2.8851	TRAF3	TNF receptor-associated factor TRAF
ENSPSIG00000005466	2.258	IGSF9	Fibronectin type III
ENSPSIG00000015424	2.2513	TNFAIP3	Zinc finger, A20-type
ENSPSIG00000014349	2.1752	PCID2	PCI/PINT associated module
ENSPSIG00000007409	2.0494	IRAK2	Death-like domain
ENSPSIG00000011862	1.5052	IGBP1	TAP42-like protein
ENSPSIG00000011812	1.382	NFAT5	Immunoglobulin E-set
ENSPSIG00000009794	1.373	TOLLIP	UBA-like
ENSPSIG00000009660	1.3213	RIPK2	Death-like domain
ENSPSIG00000016396	1.1495	SMAD1	MAD homology, MH1
down-regulated genes			
ENSPSIG00000010266	−1.0231	CTSK	cathepsin K
ENSPSIG00000007073	−1.1143	IBTK	BTB/POZ fold
ENSPSIG00000009177	−1.2203	HPGDS	Thioredoxin-like fold
ENSPSIG00000006195	−1.2357	FKBP5	Tetratricopeptide repeat
ENSPSIG00000015560	−1.264	TNFAIP2	Exocyst complex component Sec6
ENSPSIG00000014766	−1.2851	ST3GAL4	Sialyltransferase
ENSPSIG00000018025	−1.3045	IL1B	interleukin‐1β
ENSPSIG00000011723	−1.3239	IKBKG	NF-kappa-B essential modulator NEMO, N-terminal
ENSPSIG00000015425	−1.3864	NFKBIZ	Ankyrin repeat-containing domain
ENSPSIG00000009846	−1.5198	HSP90B1	Histidine kinase-like ATPase, C-terminal domain
ENSPSIG00000017778	−1.5315	PECAM1	Immunoglobulin subtype 2
ENSPSIG00000017956	−1.5466	TNFAIP8	Protein of unknown function DUF758
ENSPSIG00000007481	−1.5676	ANXA1	Annexin repeat
ENSPSIG00000010826	−1.6104	IL4R	Fibronectin type III
ENSPSIG00000000537	−1.6414	TLR2	toll-like receptor 2
ENSPSIG00000008175	−1.7424	MYD88	Toll/interleukin-1 receptor homology (TIR) domain
ENSPSIG00000007865	−1.8091	ITGB2	Plexin-like fold
ENSPSIG00000017067	−2.018	IL18	Cytokine, IL-1-like
ENSPSIG00000012636	−2.1408	IGJ	immunoglobulin J polypeptide
ENSPSIG00000012960	−2.2105	THNSL2	Tryptophan synthase subunit-like PLP-dependent enzyme
ENSPSIG00000002701	−2.337	SKAP2	SH3 domain
ENSPSIG00000013490	−2.643	DDX58	P-loop containing nucleoside triphosphate hydrolase
ENSPSIG00000007510	−2.7136	CPAMD8	Alpha-macroglobulin, receptor-binding
ENSPSIG00000007863	−2.7763	MGST2	5-lipoxygenase-activating protein
ENSPSIG00000017045	−2.8904	FKBP1B	Peptidyl-prolyl cis-trans isomerase, FKBP-type, domain
ENSPSIG00000008205	−3.2054	TNFRSF19	TNFR/NGFR cysteine-rich region
ENSPSIG00000005065	−3.4382	TNF	TNF tumor necrosis factor
ENSPSIG00000003827	−3.5594	TLR4	toll-like receptor 4
ENSPSIG00000011765	−3.5897	SFTPD	C-type lectin fold
ENSPSIG00000016170	−3.8325	IGHM	Immunoglobulin C1-set
ENSPSIG00000015877	−3.8608	IL22RA2	Fibronectin type III
ENSPSIG00000015128	−4.264	PIANP	PILR alpha associated neural protein
ENSPSIG00000003075	−4.9664	UNC93A	Major facilitator superfamily domain

**Table 3:** DEGs involved in the apoptosis pathway during sperm storage.

Gene ID	Log_2_ Fold change	Associated Gene Name	Interpro Description
up-regulated genes			
ENSPSIG00000014827	2.661	RPAP3	Tetratricopeptide repeat
ENSPSIG00000014929	2.3893	BCOR	Ankyrin repeat-containing domain
ENSPSIG00000001478	2.3731	BAG5	BAG domain
ENSPSIG00000012400	2.0129	BAG4	BAG domain
ENSPSIG00000004968	1.8734	NEK6	Protein kinase-like domain
ENSPSIG00000009078	1.7553	MCL1	Bcl2-like
ENSPSIG00000011287	1.7351	API5	Armadillo-type fold
ENSPSIG00000013461	1.7348	PDCD2	Zinc finger, MYND-type
ENSPSIG00000015104	1.645	IGF1R	Tyrosine-protein kinase, insulin-like receptor
ENSPSIG00000007435	1.5841	SIVA1	Siva
ENSPSIG00000014529	1.5404	BCL6	BTB/POZ fold
ENSPSIG00000012576	1.4298	PRKCI	Protein kinase-like domain
ENSPSIG00000004057	1.4283	GAB1	Pleckstrin homology domain
ENSPSIG00000004826	1.408	HTRA2	PDZ domain
ENSPSIG00000013535	1.381	FAF2	UAS
ENSPSIG00000004383	1.3647	TAOK1	Homeodomain-like
ENSPSIG00000006786	1.3528	ING3	Staphylcoagulase, N-terminal
ENSPSIG00000008015	1.32	PDCD6IP	BRO1 domain
ENSPSIG00000017351	1.2659	ADCK3	Protein kinase-like domain
ENSPSIG00000003952	1.2614	CCNL2	Cyclin-like
ENSPSIG00000015707	1.2495	PSMC3IP	BlaI transcriptional regulatory family
ENSPSIG00000005369	1.1682	NCOA4	Nuclear coactivator
ENSPSIG00000005191	1.1451	DIDO1	Zinc finger, FYVE/PHD-type
ENSPSIG00000015775	1.1177	OPTN	NF-kappa-B essential modulator NEMO, N-terminal
ENSPSIG00000006978	1.0971	MED1	Mediator complex, subunit Med1, metazoa/fungi
ENSPSIG00000016631	1.0249	BNIP3L	BNIP3
ENSPSIG00000017945	1.0182	CCAR2	Nucleic acid-binding, OB-fold
ENSPSIG00000004635	1.0112	NDRG1	Alpha/Beta hydrolase fold
down-regulated genes			
ENSPSIG00000016035	−0.057855	SCOTIN	P53 apoptosis protein
ENSPSIG00000008774	−1.023	PIDD	p53-induced death domain protein
ENSPSIG00000002355	−1.0367	CLDND1	PMP-22/EMP/MP20/Claudin superfamily
ENSPSIG00000004908	−1.1619	MECOM	Zinc finger, C2H2-like
ENSPSIG00000010399	−1.2458	IRF1	Interferon regulatory factor-1/2
ENSPSIG00000010376	−1.5406	MSX2	Homeodomain-like
ENSPSIG00000004029	−1.5816	CASP9	apoptosis-related cysteine peptidase
ENSPSIG00000012821	−1.6969	ELMO2	Armadillo-type fold
ENSPSIG00000017598	−1.7528	FOS	Transcription factor, Skn-1-like, DNA-binding domain
ENSPSIG00000005216	−1.7926	AHSA1	Activator of Hsp90 ATPase, N-terminal
ENSPSIG00000017957	−1.8668	CIAPIN1	S-adenosyl-L-methionine-dependent methyltransferase
ENSPSIG00000002892	−1.9317	CytC	cytochrome c-like
ENSPSIG00000003046	−2.0734	CASP6	Peptidase C14A, caspase precursor p45, core
ENSPSIG00000008544	−2.0876	PARM1	prostate androgen-regulated mucin-like protein
ENSPSIG00000015307	−2.23	BCL2L11	Apoptosis, Bim N-terminal
ENSPSIG00000008949	−3.3761	CARD10	Death-like domain
ENSPSIG00000009528	−3.3981	DTHD1	Death-like domain
ENSPSIG00000016980	−5.0398	SFRP4	Tissue inhibitor of metalloproteinases-like, OB-fold
ENSPSIG00000015419	−6.2964	PERP	TP53 apoptosis effector

**Table 4:** DEGs involved in the regulation of autophagy during sperm storage.

Gene ID	Log_2_ Fold change	Associated Gene Name	Interpro Description
up-regulated genes			
ENSPSIG00000012704	2.9702	MAP1LC3B	Ubiquitin-related domain
ENSPSIG00000002664	2.771	ATG5	Autophagy-related protein 5
ENSPSIG00000006041	2.1118	CALCOCO2	Coiled-coil transcriptional coactivator-like
ENSPSIG00000015566	2.0203	ULK2	Protein kinase-like domain
ENSPSIG00000010882	1.8742	ATG14	UV radiation resistance protein/autophagy-related protein 14
ENSPSIG00000006840	1.6022	SH3GLB1	SH3 domain
ENSPSIG00000009647	1.5666	SQSTM1	UBA-like
ENSPSIG00000002171	1.5491	ATG16L1	STAT transcription factor, coiled coil
ENSPSIG00000017272	1.5223	ATG12	Ubiquitin-related domain
ENSPSIG00000015127	1.2743	RB1CC1	Ubiquitin-related domain
ENSPSIG00000016631	1.0249	BNIP3	BNIP3
down-regulated genes			
ENSPSIG00000005809	−1.0766	STX5	t-SNARE
ENSPSIG00000016695	−1.6898	TECPR2	Regulator of chromosome condensation 1/beta-lactamase-inhibitor protein II
ENSPSIG00000008902	−2.7236	VMP1	vacuole membrane protein 1
