# Supplementary material for: Global analysis of differential gene expression related to long-term sperm storage in oviduct of Chinese Soft-Shelled Turtle Pelodiscus sinensis
Source: Sci Rep. 2016 Sep 15;6:33296. doi: 10.1038/srep33296 (PMC5024102; doi:10.1038/srep33296)
Supplement: Supplementary Information [file srep33296-s1.pdf]

Supporting information

Global analysis of differential gene expression related to long-term sperm storage in oviduct of Chinese Soft-Shelled Turtle *Pelodiscus sinensis*

Tengfei Liu[1], Ping Yang[1], Hong Chen[1], Yufei Huang[1], Yi Liu[1], Yasir Waqas[1], Nisar Ahmed[1], Xiaoya Chu[1], Qiusheng Chen[1]*

Supporting information

Supplementary Fig. S1 Western blot analysis of LC3B in the *P. sinensis* oviduct and rat liver with the positive control. The negative controls were obtained by exchanging the primary antibody with 0.01 M PBS.

Supplementary Fig. S2 The identified sperm storage-related DEGs involved in Toll-like receptor (TLR) signalling pathway (A), apoptosis pathway (B), p53 signalling pathway (C), and regulation of autophagy pathway (D). These pathways originate from Kyoto Encyclopedia of Genes and Genome (KEGG) database. The red and green colors indicate up- and down-regulated genes, respectively.

Supplementary Table S1 Enriched GO terms of up-regulated DEGs in *P. sinensis*

Supplementary Table S2 Enriched GO terms of down-regulated DEGs in *P. sinensis*

Supplementary Table S3 Significantly enriched pathways of significantly DEGs in *P. sinensis*

Supplementary Table S4 Detailed information of DEGs related to sperm storage in *P. sinensis*

Supplementary Table S5 Primers of DEGs for RT-qPCR analysis in *P. sinensis*

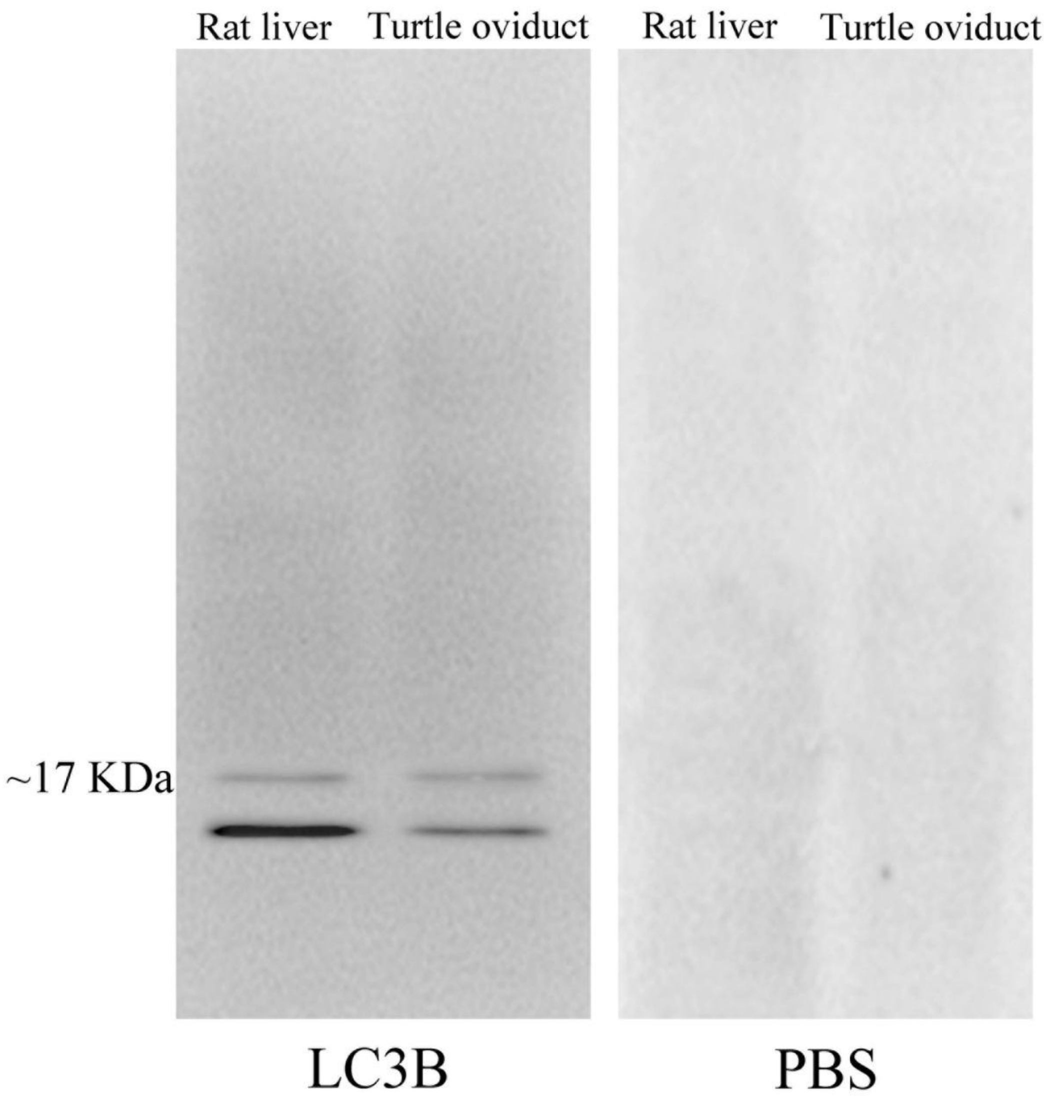

Supplementary Fig. S1 Western blot analysis of LC3B in the *P. sinensis* oviduct and rat liver with the positive control. The negative controls were obtained by exchanging the primary antibody with 0.01 M PBS.

A

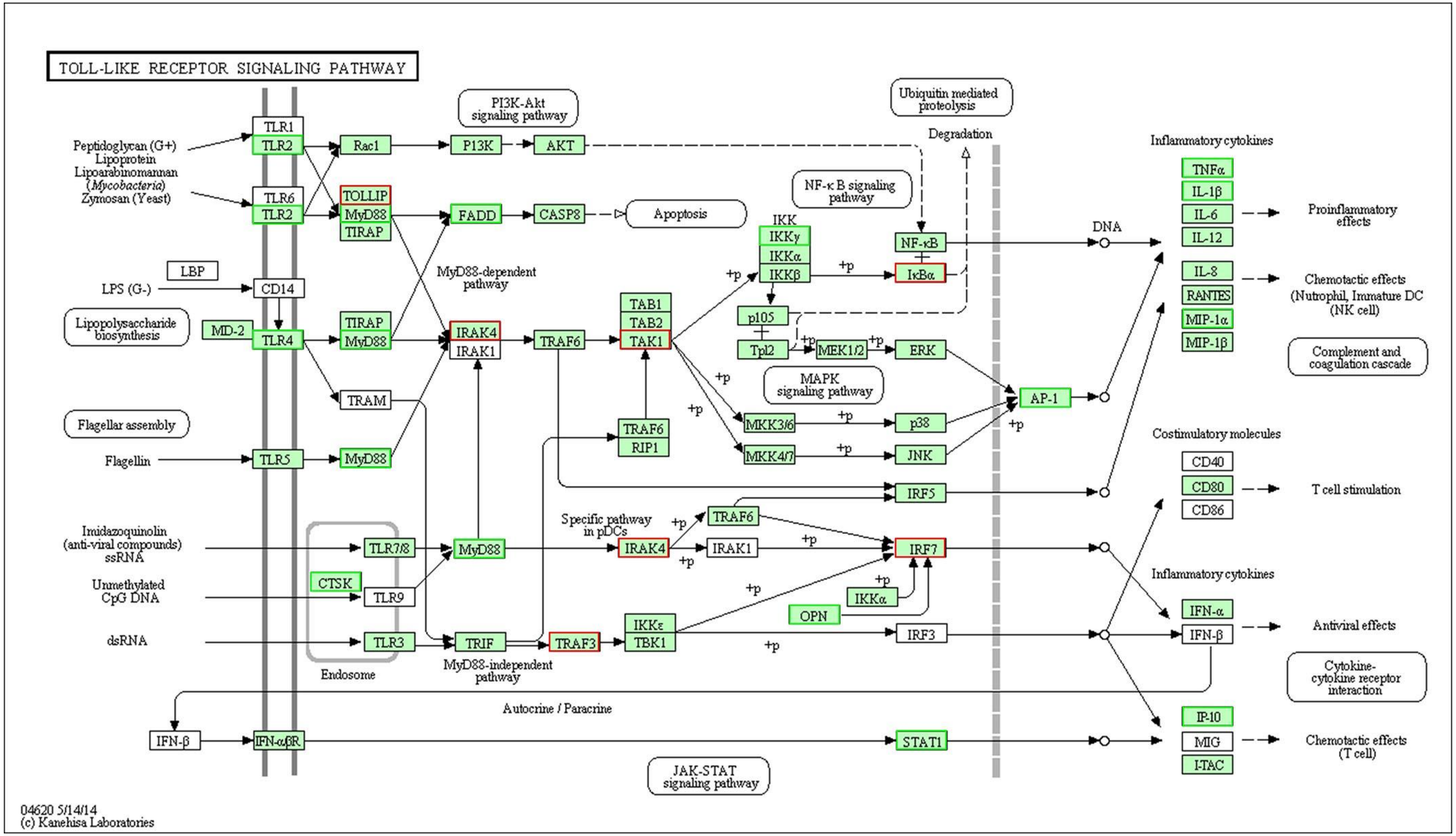

TOLL-LIKE RECEPTOR SIGNALING PATHWAY
Peptidoglycan (G+)
Lipoprotein
Lipoarabinomannan
(Mycobacteria)
Zymosan (Yeast)
TLR1
TLR2
TLR6
TLR2
Rac1
PI3K
AKT
PI3K-Akt signaling pathway
TOLLIP
MyD88
TIRAP
FADD
CASP8
Apoptosis
LBP
LPS (G-)
CD14
Lipopolysaccharide biosynthesis
MD-2
TLR4
TIRAP
MyD88
MyD88-dependent pathway
IRAK4
IRAK1
TRAF6
TAB1
TAB2
TAK1
TRAM
Flagellar assembly
Flagellin
TLR5
MyD88
TRAF6
RIP1
Ubiquitin mediated proteolysis
Degradation
NF-κB signaling pathway
IKK
IKKγ
IKKα
IKKβ
NF-κB
IκBα
p105
Tpl2
MEK1/2
ERK
MAPK signaling pathway
MKK3/6
p38
MKK4/7
JNK
AP-1
IRF5
DNA
+p
Imidazoquinolin
(anti-viral compounds)
ssRNA
Unmethylated
CpG DNA
dsRNA
TLR7/8
CTSK
TLR9
TLR3
Endosome
MyD88
Specific pathway in pDCs
IRAK4
IRAK1
TRAF6
IRF7
IKKα
OPN
TRIF
TRAF3
IKKε
TBK1
IRF3
MyD88-independent pathway
Autocrine / Paracrine
IFN-β
IFN-αβR
STAT1
JAK-STAT signaling pathway
Inflammatory cytokines
TNFα
IL-1β
IL-6
IL-12
Proinflammatory effects
IL-8
RANTES
MIP-1α
MIP-1β
Chemotactic effects
(Nutrophil, Immature DC
(NK cell)
Complement and coagulation cascade
Costimulatory molecules
CD40
CD80
CD86
T cell stimulation
Inflammatory cytokines
IFN-α
IFN-β
Antiviral effects
Cytokine-cytokine receptor interaction
IP-10
MIG
I-TAC
Chemotactic effects
(T cell)
04620 5/14/14
(c) Kanehisa Laboratories

B

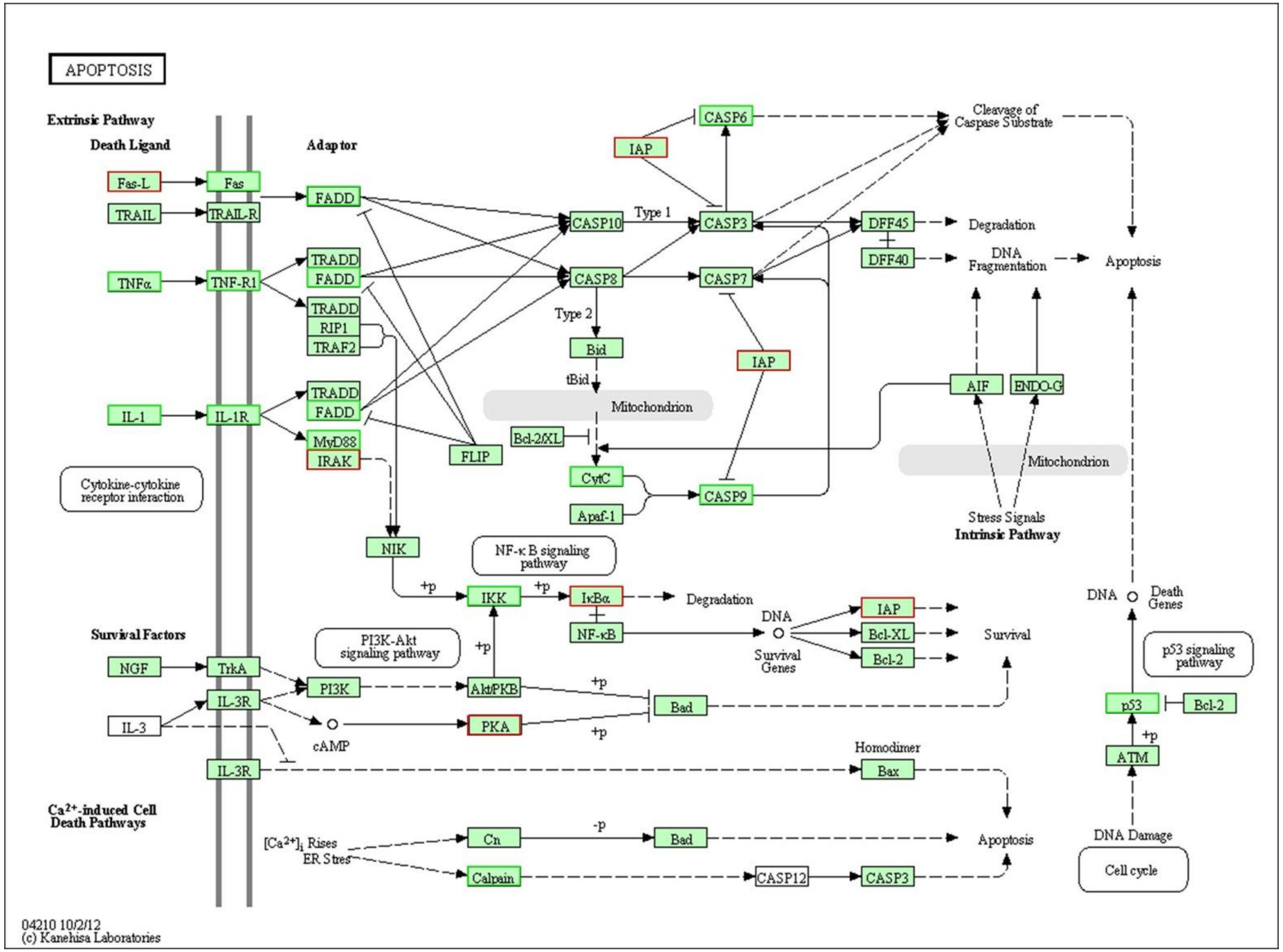
APOPTOSIS
Extrinsic Pathway
Death Ligand
Adaptor
Fas-L
Fas
TRAIL
TRAIL-R
FADD
TNFα
TNF-R1
TRADD
FADD
TRADD
RIP1
TRAF2
IL-1
IL-1R
TRADD
FADD
MyD88
IRAK
FLIP
CASP10
CASP8
Type 1
Type 2
CASP3
CASP7
CASP6
IAP
IAP
Cleavage of Caspase Substrate
DFF45
DFF40
Degradation
DNA Fragmentation
Apoptosis
Bid
tBid
Mitochondrion
Bcl-2/XL
CytC
Apaf-1
CASP9
AIF
ENDO-G
Mitochondrion
Stress Signals
Intrinsic Pathway
Cytokine-cytokine receptor interaction
NIK
NF-κB signaling pathway
+p
IKK
+p
IκBα
Degradation
NF-κB
DNA
Survival Genes
IAP
Bcl-XL
Bcl-2
Survival
Survival Factors
NGF
TrkA
PI3K-Akt signaling pathway
IL-3R
PI3K
Akt/PKB
+p
IL-3
cAMP
PKA
+p
+p
Bad
IL-3R
Homodimer
Bax
Apoptosis
Ca2+-induced Cell Death Pathways
[Ca2+]i Rises
ER Stres
Cn
-p
Bad
Calpain
CASP12
CASP3
DNA
Death Genes
p53 signaling pathway
p53
Bcl-2
+p
ATM
DNA Damage
Cell cycle
04210 10/2/12
(c) Kanehisa Laboratories

C

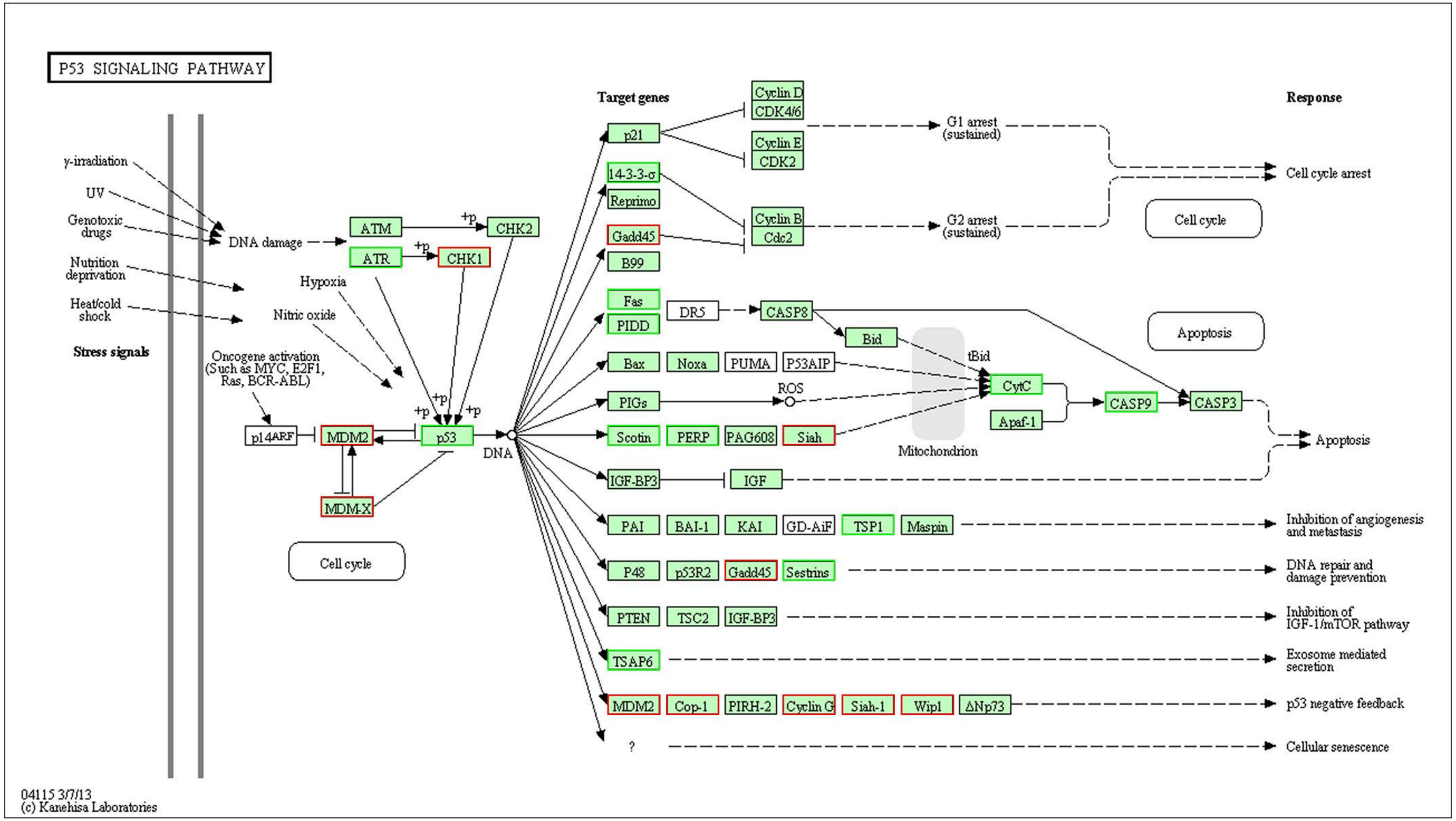

P53 SIGNALING PATHWAY
γ-irradiation
UV
Genotoxic drugs
Nutrition deprivation
Heat/cold shock
Stress signals
DNA damage
ATM
ATR
CHK2
CHK1
+p
Hypoxia
Nitric oxide
Oncogene activation (Such as MYC, E2F1, Ras, BCR-ABL)
p14ARF
MDM2
MDM-X
p53
DNA
Cell cycle
Target genes
p21
14-3-3-σ
Reprimo
Gadd45
B99
Fas
PIDD
DR5
CASP8
Bid
tBid
Bax
Noxa
PUMA
P53AIP
CytC
Apaf-1
CASP9
CASP3
PIGs
ROS
Scotin
PERP
PAG608
Siah
Mitochondrion
IGF-BP3
IGF
Cyclin D
CDK4/6
Cyclin E
CDK2
Cyclin B
Cdc2
G1 arrest (sustained)
G2 arrest (sustained)
Cell cycle
Apoptosis
PAI
BAI-1
KAI
GD-AiF
TSP1
Maspin
P48
p53R2
Gadd45
Sestrins
PTEN
TSC2
IGF-BP3
TSAP6
MDM2
Cop-1
PIRH-2
Cyclin G
Siah-1
Wip1
ΔNp73
?
Response
Cell cycle arrest
Apoptosis
Inhibition of angiogenesis and metastasis
DNA repair and damage prevention
Inhibition of IGF-1/mTOR pathway
Exosome mediated secretion
p53 negative feedback
Cellular senescence
04115 3/7/13
(c) Kanehisa Laboratories

D

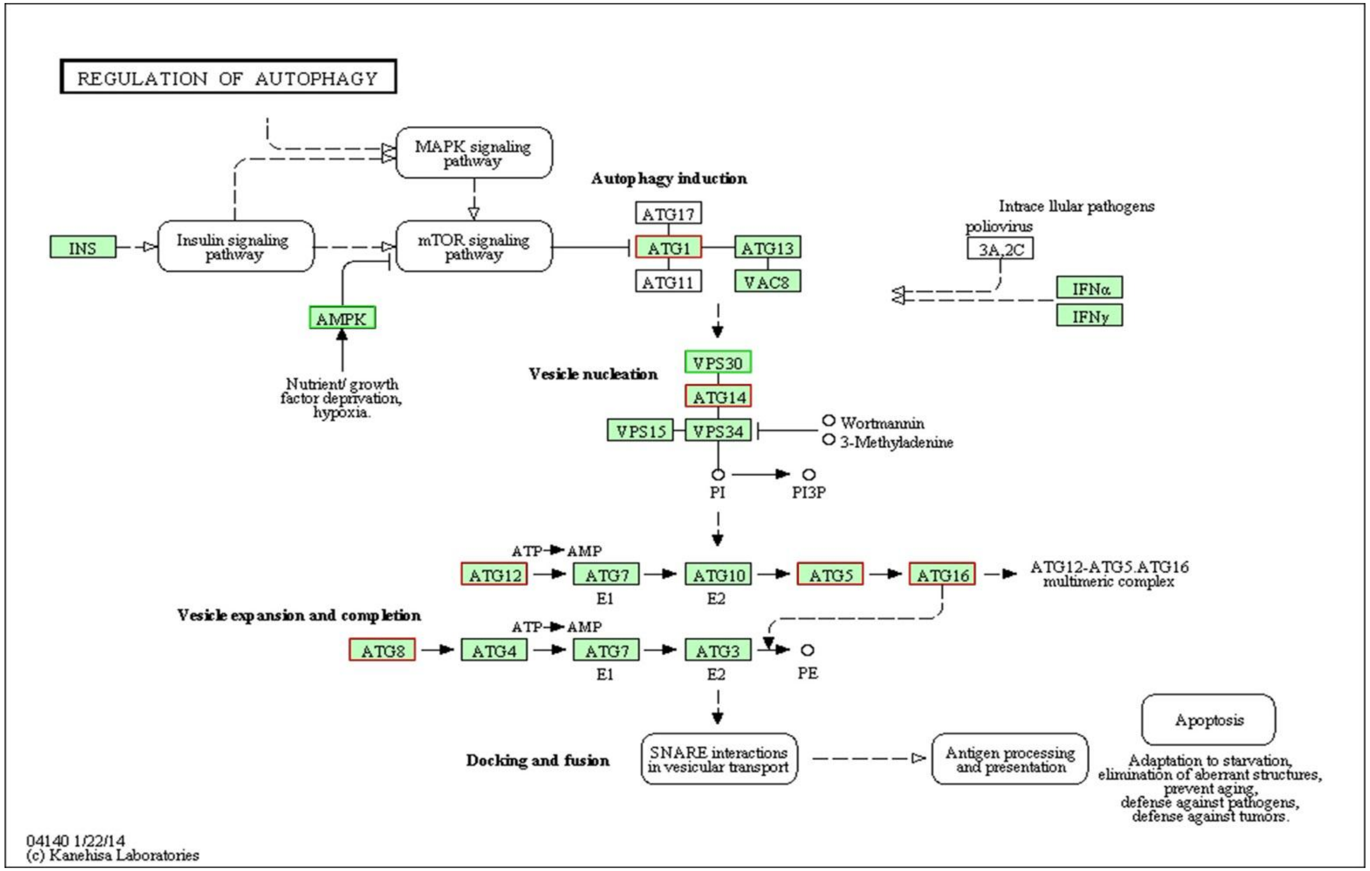

Supplementary Fig. S2 The identified sperm storage-related DEGs involved in Toll-like receptor (TLR) signalling pathway (A), apoptosis pathway (B), p53 signalling pathway (C), and regulation of autophagy pathway (D). These pathways originate from Kyoto Encyclopedia of Genes and Genome (KEGG) database. The red and green colors indicate up- and down-regulated genes, respectively.
